# Museum of the Baltimore College of Dental Surgery

**Published:** 1845-12

**Authors:** 


*Museum of the Baltimore College of Dental Surgery.*—A very valuable contribution was made to the Museum of this Institution a few weeks since by Dr. John Harris, consisting of between twelve and fifteen thousand morbid dental specimens. Similar contributions would be thankfully received by the Faculty, from other members of the profession.

